# Supplementary material for: Beyond the Virus: The Collateral Impact of COVID-19 on Antimicrobial Consumption, Microbial Resistance, and Pharmacoeconomics
Source: Pathogens. 2025 Nov 5;14(11):1126. doi: 10.3390/pathogens14111126 (PMC12654989; doi:10.3390/pathogens14111126)
Supplement: Supplementary file 1 [file pathogens-14-01126-s001.zip › pathogens-3936249-supplementary.pdf]

Beyond the Virus: The Collateral Impact of COVID-19 on Antimicrobial Consumption, Microbial Resistance, and Pharmacoeconomics

Supplemental Material

Alessandra Gomes Chauvin[1*], Isabele Pardo[2], André Luis F. Cotia[1], Isabella L. Rosmino[1], Tatiana A. Marins[1], Leandro Martins dos Santos[1], Barbara Barduchi[1], Alexandra R. Toniolo[1], Roberta G. dos Santos[1], Daniel T. Malheiro[1], Anderson P. Scorsato[1], Elivane da Silva Victor[1]; Michael B. Edmond[4], Silvana Maria de Almeida[1] and Alexandre R. Marra[1,3]

[1] Hospital Israelita Albert Einstein, São Paulo, SP, Brazil - 05652-900; alexandre.marra@einstein.br (A.R.M.); andre.cotia@einstein.br (A.L.F.C.); isabella.rosmino@einstein.br (I.L.R.); tatiana.marins@einstein.br (T.A.M.); leandro.msantos@einstein.br (L.M.d.S.); barbara.barduchi@einstein.br (B.B.); alexandra.toniolo@einstein.br (A.R.T.); roberta.gsantos@einstein.br (R.G.d.S.); daniel.malheiro@einstein.br (D.T.M.); anderson.scorsato@einstein.br (A.P.S.); elivane.victor@einstein.br (E.S.V.); silvana.almeida@einstein.br (S.M.d.A.)

[2] Faculdade Israelita de Ciências da Saúde Albert Einstein, Hospital Israelita Albert Einstein, São Paulo, SP, Brazil - 05652-900; isabele.pardo@einstein.edu.br (I.P.)

[3] Department of Internal Medicine, University of Iowa Health Care, Iowa City, IA - 52242-1007, USA; alexandre-rodriguesmarra@uiowa.edu (A.R.M.)

[4] Department of Medicine, School of Medicine, West Virginia University, Morgantown, WV - 26506-9111, USA; michael.edmond1@wvumedicine.org (M.B.E.)

* Correspondence: alessandra.chauvin@einstein.br (A.G.C.); Tel.: +55-(67)-98421-0187

1. Contents

1. **Cover Page** ……………….……......………………...1

2. **Contents** ………………………..………...2

3. **Figure S1**. Time series of mean DDD of the carbapenems group…………..…..3

4. **Figure S2**. Time series of mean DDD of the glycopeptides group……..………..3

5. **Figure S3**. Time series of mean DDD of the polymyxins group…………………3

6. **Table S1.** Comparison of antimicrobial consumption (DOT) per 1000 patient-days between the periods before and during COVID-19…………………………4

7. **Figure S4**. Heatmap chart of antimicrobial adverse reactions before and during COVID-19………………………..………...5

8. **Table S2.** Linear regression model (DDD Carbapenem Group) 5

9. **Table S3.** Linear regression model (DDD Glycopeptides Group) 5

10. **Table S4.** Linear regression model (DDD Macrolides Group) 6

11. **Table S5**. Linear regression model (DDD Polymyxins Group) 6

12. **Table S6.** Linear regression model (DDD Echinocandins Group) 6

13. **Table S7**. Linear regression model (DDD Antivirals Group) 6

Figure S1. Time series of mean DDD of the carbapenems group

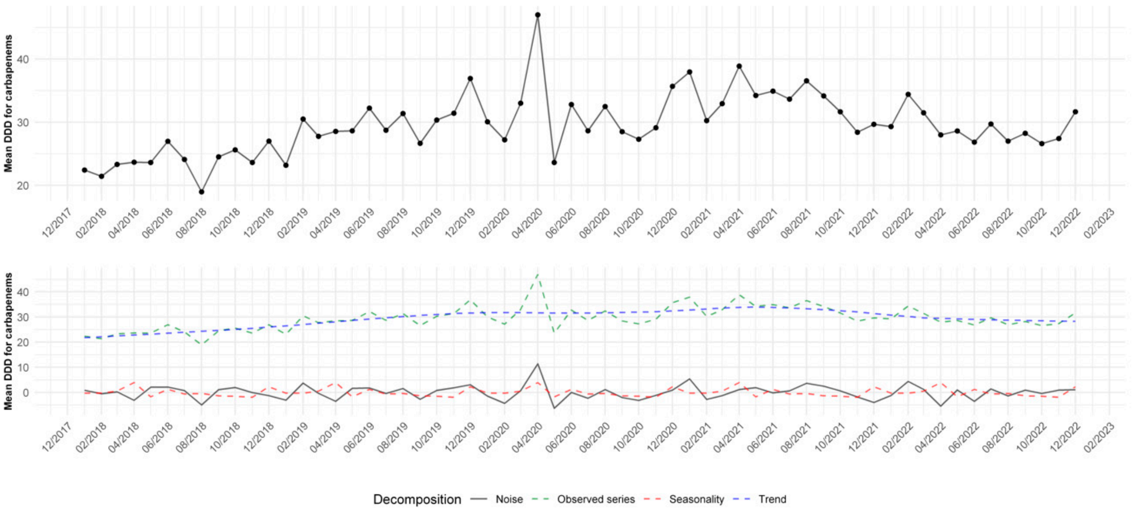

Figure S2. Time series of mean DDD of the glycopeptides group

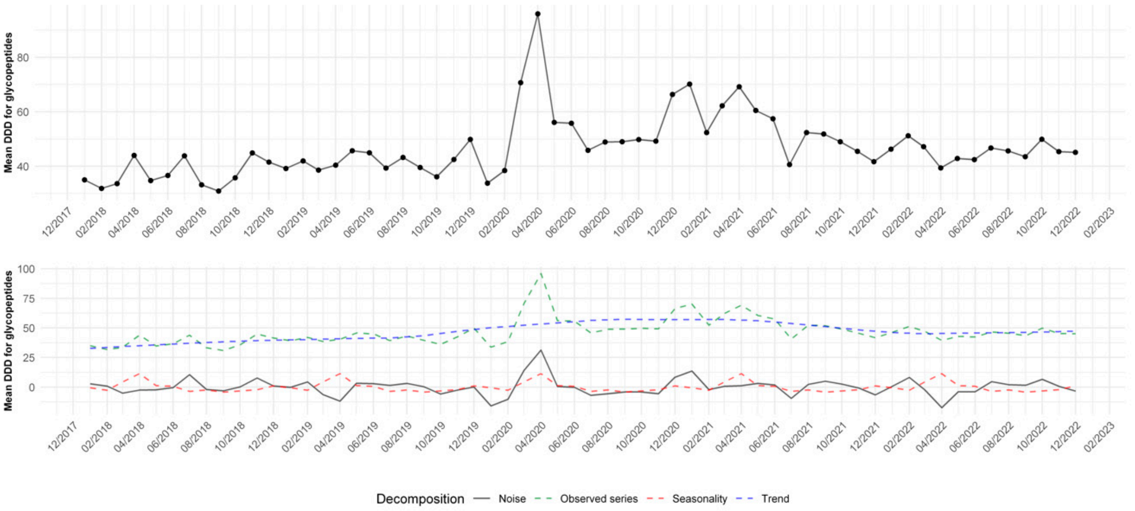

Figure S3. Time series of mean DDD of the polymyxins group.

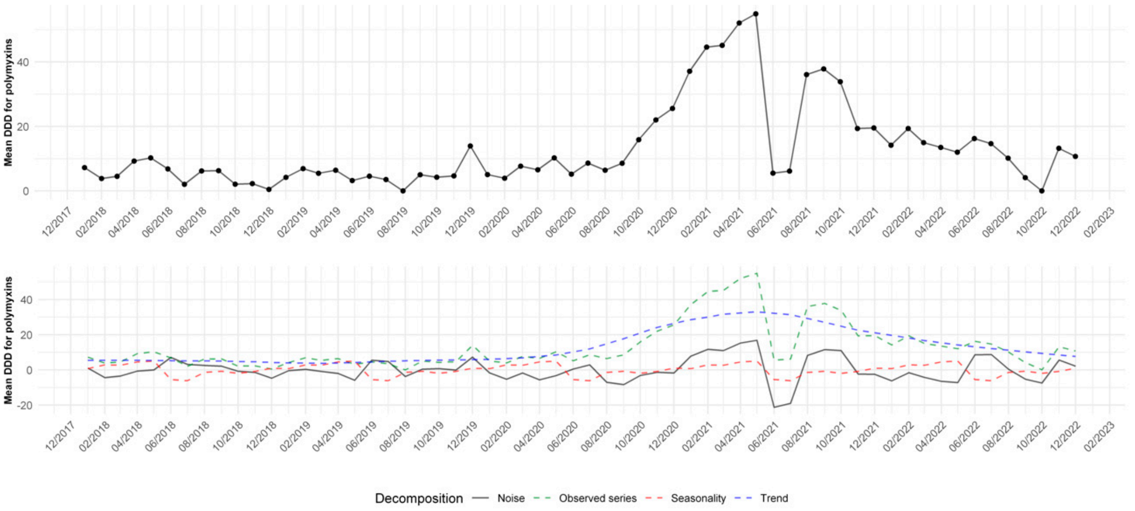

Table S1. Comparison of antimicrobial consumption (DOT) per 1000 patient-days between the periods before and during COVID-19

Antimicrobials	Before COVID-19 (Mean ± SD)	During COVID-19 (Mean ± SD)	p-value
**1st and 2nd generation cephalosporin**			
Mean ± SD	75.3 ± 5.0	64.4 ± 12.7	
Median [IIQ]	75.3 [73.2 - 80.0]	68.5 [58.3 - 73.8]	<0.001
**3rd, 4th and 5th cephalosporin**			
Mean ± SD	39.1 ± 2.5	45.7 ± 8.2	
Median [IQR]	38.7 [37.4 - 40.7]	43.3 [39.2 - 50.6]	<0.001
**Cephalosporin + β-lactamase inhibitors**			
Mean ± SD	1.7 ± 2.0	11.9 ± 3.6	
Median [IQR]	0.6 [0 - 3.2]	11.8 [9.1 - 14.1]	<0.001
**Macrolides**			
Mean ± SD	13.6 ± 2.3	18.7 ± 9.9	
Median [IQR]	13.1 [12.3 - 14.9]	16,4 [10.6 - 22.3]	0.143
**Carbapenems**			
Mean ± SD	32.7 ± 4,6	38.1 ± 5.3	
Median [IQR]	31.5 [29.2 - 34.7]	38.3 [35.0 - 40.7]	<0.001
**Glycopeptides**			
Mean ± SD	38.7 ± 5.7	53.2 ± 8.1	
Median [IQR]	39.1 [33.8 - 42.9]	50.7 [47.8 - 55.3]	<0.001
**Polymyxin B**			
Mean ± SD	4.4 ± 1.7	13.8 ± 10.1	
Median [IQR]	4.3 [3.61 – 5.4]	11.12 [6.79 – 17.9]	<0.001
**Echinocandins**			
Mean ± SD	9.6 ± 2.3	14.1 ± 4.2	*<0.001
Median [IQR]	9,8 [8.1 – 11.5]	13.5 [10.7 – 16.7]	
**Triazole Antifungals**			
Mean ± SD	1.3 ± 2.3	12.2 ± 2.4	*0.164
Median [IQR]	11.6 [9.5 – 13.2]	12.1 [10.4 - 13.4]	
**Antivirals**			
Mean ± SD	8.9 ± 3.3	5.9 ± 3.6	
Median [IQR]	8.0 [6.4 - 9.8]	4.8 [3.2 - 7.5]	<0.001

Note: IQR: Interquartile range (1st and 3rd quartiles); SD: Standard deviation

Figure S4. Heatmap chart of antimicrobial adverse reactions before and during COVID-19

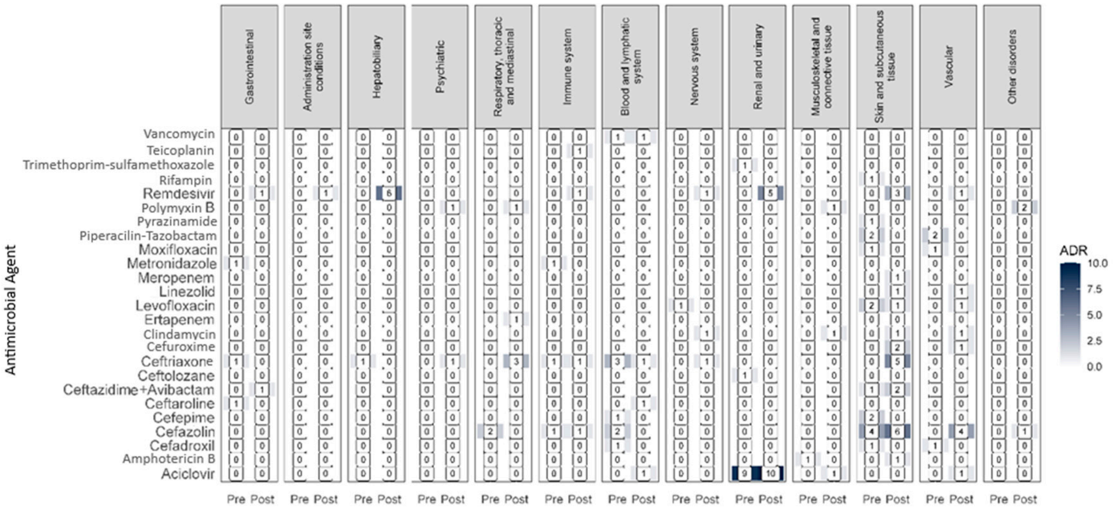

Table S2. Linear regression model (DDD Carbapenem Group)

WLS regression	Estimate	Standard error	T value	p-value
**(Intercept)**	41.330	3.231	12.792	-
**Total patient-days in the month**	-0.005	0.001	-6.39	<0.001
**Total Cost (per 1,000,000 US$)**	0.060	0.009	6.962	<0.001
**BSI rate (per 1,000 central line-days)**	-2.928	1.393	-2.103	0.040
**VAP-SDU rate in the step-down unit (per 1,000 ventilator-days)**	-4.514	1.546	-2.919	0.005

Model diagnostics: Durbin–Watson test (lag 1): p = 0.166 / Durbin–Watson test (lag 4): p = 0.006 / Breusch–Pagan test (heteroscedasticity): p = 0.566

Abbreviations: WLS: weighted least squares; DDD: defined daily dose; BSI = bloodstream infection; VAP-SDU = ventilator-associated pneumonia in the step-down unit

Table S3. Linear regression model (DDD Glycopeptides Group)

WLS regression (with 2nd differencing)	Estimate	Standard error	T value	p-value
**(Intercept)**	80.780	12.720	6.35	-
**Total patient-days in the month**	-0.013	0.002	-5.457	<0.001
**Total Costs (per 1,000,000 US$)**	0.136	0.024	5.568	<0.001
**First difference**	0.167	0.117	1.435	0.157
**Second difference**	-0.253	0.121	-2.089	0.042

Model diagnostics: Durbin–Watson test (lag 1): p = 0.224 / Durbin–Watson test (lag 2): p = 0.412/ Breusch–Pagan test (heteroscedasticity): p = 0.983

Abbreviations: WLS: weighted least squares; DDD: defined daily dose

Table S4. Linear regression model (DDD Macrolides Group)

WLS regression	Estimate	Standard error	T value	p-value
**(Intercept)**	60.020	7.624	7.872	-
**Total patient-days in the month**	-0.009	0.002	-5.290	<0.001
**COVID-19 deaths**	0.318	0.122	2.611	0.012
**Adverse Drug Reactions**	0.147	0.082	1.797	0.078

Model diagnostics: Durbin–Watson test (lag 1): p = 0.090 / Breusch–Pagan test (heteroscedasticity): p = 0.989

Abbreviations: WLS: weighted least squares; DDD: defined daily dose

Table S5. Linear regression model (DDD Polymyxins Group)

WLS regression (with 2nd differencing)	Estimate	Standard error	T value	p-value
**(Intercept)**	2.723	0.9853	2.763	-
**COVID-19 deaths**	0.292	0.1128	2.590	0.012
**First difference**	0.701	0.1174	5.972	<0.001
**Second difference**	-0.134	0.1130	-1.189	0.240

Model diagnostics: Durbin–Watson test (lag 1): p = 0.928 / Durbin–Watson test (lag 2): p = 0.090/ Breusch–Pagan test (heteroscedasticity): p = 0.999

Abbreviations: WLS: weighted least squares; DDD: defined daily dose

Table S6. Linear regression model (DDD Echinocandins Group)

WLS regression (with 1st differencing)	Estimate	Standard error	T value	p-value
**Total patient-days in the month**	-0.005	0.001	-3.150	0.003
**Total Costs (per 1,000,000 US$)**	0.073	0.019	3.929	<0.001
**Pharmaceutical activities (per 1,000 pharmacists)**	0.849	0.422	2.010	0.050
**Multidrug-resistant organism**	1.977	0.636	3.110	0.003
**ESKAPE**	-2.168	0.635	-3.412	0.001
**First difference**	0.419	0.122	3.437	0.001

Model diagnostics: Durbin–Watson test (lag 1): p = 0.190 / Breusch–Pagan test (heteroscedasticity): p = 0.958

Abbreviations: WLS: weighted least squares; DDD: defined daily dose

Table S7. Linear regression model (DDD Antivirals Group)

WLS regression (with 1st differencing)	Estimate	Standard error	T value	p-value
**(Intercept)**	4.497	3.897	1.154	-
***Clostridioides difficile* incidence rate**	0.5371	0.2657	2.021	0.048
**Total Cost (per 1,000,000 US$)**	-0.027	0.016	-1.641	0.107
**Adverse Drug Reactions**	0.1944	0.1001	1.941	0.057

**First difference**	0.09774	0.08501	1.15	0.255

Model diagnostics: Durbin–Watson test (lag 1): p = 0.022 / Breusch–Pagan test (heteroscedasticity): p = 0.999
